# Nitrate of Silver in Root-Canals

**Published:** 1897-02

**Authors:** Charles D. Cheney

**Affiliations:** Hoboken, N. J.


### NITRATE OF SILVER IN ROOT-CANALS.

BY CHARLES D. CHENEY, HOBOKEN, N. J.

The publication in your November issue of a paper upon a nitrate of silver method of root-canal treatment decides me to give my own experience in the same direction.

I would state that I have been experimenting (if a steady reliance may be so designated) with silver nitrate for over two years. I have used it in root-canals during about that period, and have come to believe that in it we have as near an ideal as we are likely to get.

I have not before this publication seen or heard silver nitrate suggested for root-canal treatment; it was, however, but a short step for me from the use of it in an ordinary cavity of decay to use in the canals. I have used it with satisfaction in ordinary cavities for a number of years, and during the period of *canal* use my satisfaction has been infinitely increased.

I may say I consider pulp-extirpation and canal-filling more scientific than any method of so-called pulp-"capping," especially since silver nitrate makes the former operation practically successful and certain.

I have never seen any objectionable effects from its use in nerve-canals, or I believe I may say *any* "effects." The use of the nitrate when the pulp is but thinly protected by dentine is not to be tolerated, but I have not observed any irritation to the pericementum when the canal may have been large and the walls thin.

An important improvement which I very early discovered and have used exclusively since was the substitution of an alcoholic saturated solution of silver nitrate for the customary aqueous solution. This is an improvement which I consider essential to thorough success. Alcohol dissolves less of the nitrate, but the rapid evaporation compensates and makes a less quantity equally efficacious; the alcohol also penetrates farther and quicker than water; a warm-air blast or even a few moments' delay and the natural body heat gets rid of the solvent, and also any small degree of moisture which may be present, thus leaving a deposit of microscopic nitrate crystals to continue the effect very slowly as the natural moisture returns to the immediate vicinity.

Rather strange, it seems, but there appears to be within the canals none of the usual discoloration of silver nitrate, except in the presence of organic decomposition, when the precipitation of black oxide or sulphide is instantaneous. Decayed dentine is not so affected, though it often becomes slightly yellowed.

The silver nitrate seems to take such thorough care of affairs within the canal that it would seem less important what filling is used, provided it be non-absorbent and unchangeable. I have good success with gutta-percha, avoiding most religiously, however, all that which has any considerable "toughness,"—that is, a quality which is incompatible with its successful use in any way.

The painting of the surrounding gum with strong tincture of iodine, immediately after the filling, contributes in a large degree towards success and the comfort of the tooth, as I believe.

Occasionally the slight soreness due to the operation may persist over twenty-four hours, in which case I repeat the application of iodine or advise capsicum plasters, according to circumstances.

